# Sports Injuries: Misinterpretations to Learn From

**DOI:** 10.5334/jbr-btr.1207

**Published:** 2016-11-19

**Authors:** Reto Sutter

**Affiliations:** 1 Balgrist University Hospital, Zurich, Switzerland

## Abstract

Athletes present with a variety of both common and specific injuries to the sports physician. For the imaging evaluation of such injuries, this poses special problems to avoid misinterpretations. While some sports injuries are not complicated, other are initially misdiagnosed, leading to possible secondary complications. Further, seemingly abnormal imaging findings in athletes can actually be normal physiological and mechanical phenomenons. Especially in young athletes, a variety of pitfalls are encountered at imaging, such as a focal periphyseal edema or a cortical desmoid. Good communication between the sports physician and the radiologist is paramount in reaching a fast and correct diagnosis in such cases.

In patients with sports injuries, some diagnoses are straightforward and well-known. Others are difficult and only correctly diagnosed and treated after a delay of weeks or months.

This can lead to complications in the mid- and long term. As an example, in a young athlete with an acute tear of the anterior cruciate ligament of the knee, there is often hematoma in the soft tissues surrounding the joint capsule. In such patients, injuries to the joint capsule and the posterolateral or posteromedial stabilizing structures may be missed – which can lead to a posterolateral or posteromedial instability of the joint if left untreated [1]. This shows how important it is to reach the correct diagnosis as soon as possible, because this delay and secondary complications may prevent the athlete from competing at an elite level. Moreover, there are a number of typical injury patterns in athletes that are rarely seen in nonathletes, such as a fracture of the lateral process of the talus, the so-called snowboarder’s ankle [2]. If the treating physicians have no knowledge about the specific injury patterns in the athlete, there is a risk of misinterpretation and subsequent false therapy for these patients.

On the other hand, there are a number of seemingly abnormal findings in athletes detected at imaging that are commonly encountered but are not pathological. Plantar fasciitis is a common problem of running athletes and can be detected at MRI with increased signal intensity in the plantar fascia, and in chronic cases even by bone spurs on radiographs. However, many asymptomatic individuals have similar findings on radiographs and MRI. At MRI, for instance, a fifth of asymptomatic healthy individuals show T1-hyperintense signal of the plantar fascia at its origin, and in fluid-sensitive sequences, 4–5 percent of asymptomatic healthy individuals show hyperintense signal [3]. Furthermore, about a fifth of asymptomatic individuals show some soft-tissue edema adjacent to the origin of the plantar fascia.

In the developing skeleton, there are a number of findings that are often misinterpreted. One of these findings is an abundance of small foci of red bone marrow dispersed over the bones of the hindfoot that is commonly seen at MRI. While beginners may mistake this for a sign of systemic disease, this should be recognized as a normal finding. Other lesions are seen in the knee, such as the focal periphyseal edema (FOPE) that is thought to arise in the early stages of the physiologic closure of the physis [4]. These edematous zones are commonly seen in both young girls and boys between the ages of 12 and 15 years and may be painful. Nevertheless, this is a transitory phenomenon that does not need further evaluation.

Independent from the age of the athletes, there are a number of pseudo-tumors that can be recognized at imaging, such as chronic traction injuries at the origin of the gastrocnemius muscle at the distal femur, the so-called cortical desmoid [5]. This can be misinterpreted as an aggressive osseous neoplasia, as it is often associated with both bone marrow edema and surrounding soft tissue edema. Some patients are also referred to the radiology department with suspected tumor (Figure 1), such as young soccer players with swelling in the groin area caused by an avulsion injury of the rectus femoris origin at the anterior inferior iliac spine [6].

**Figure 1:**
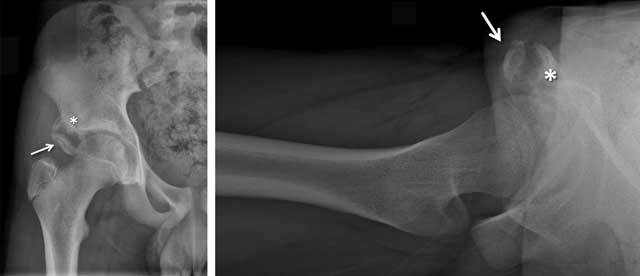
Anteroposterior and cross-table lateral radiograph of the right hip joint in a 15-year-old male soccer player who presented with a painful swelling and suspected neoplasia of the right hip region. Both the avulsed osseous fragment (arrow) and the osseous irregularities at the anterior inferior iliac spine (asterisk) are well visualized, as this was not a tumor but an avulsion injury at the origin of the rectus femoris muscle.

In runners, tibial stress fractures and the medial tibial stress syndrome are common entities, which are associated with thickening of the cortical bone, and some amounts of bone marrow edema and periosteal edematous reaction [7]. In cases with only a short fracture line, the cortical changes can be misinterpreted as osteoid osteoma. This shows the need for good communication between the referring sports physician and the radiologist, as the patient history and clinical examination can help in avoiding the misinterpretation of the imaging findings.

## References

[1] Pedersen RR (2016). The medial and posteromedial ligamentous and capsular structures of the knee: Review of anatomy and relevant imaging findings. Seminars in Musculoskeletal Radiology.

[2] Kirkpatrick DP, Hunter RE, Janes PC, Mastrangelo J, Nicholas RA (1998). The snowboarder’s foot and ankle. Am J Sports Med.

[3] Ehrmann C, Maier M, Mengiardi B, Pfirrmann CW, Sutter R (2014). Calcaneal attachment of the plantar fascia: MR findings in asymptomatic volunteers. Radiology.

[4] Zbojniewicz AM, Laor T (2011). Focal periphyseal edema (FOPE) zone on MRI of the adolescent knee: A potentially painful manifestation of physiologic physeal fusion?. AJR Am J Roentgenol.

[5] Vieira RL, Bencardino JT, Rosenberg ZS, Nomikos G (2011). MRI features of cortical desmoid in acute knee trauma. AJR Am J Roentgenol.

[6] Sutter R, Pfirrmann CWA (2013). Atypical hip impingement. Am J Roentgenol.

[7] Franklyn M, Oakes B (2015). Aetiology and mechanisms of injury in medial tibial stress syndrome: Current and future developments. World J Orthop.

